# Evaluation of the Behavioral Determinants of Infant Oral Hygiene Practices in a Rural Area

**DOI:** 10.7759/cureus.40550

**Published:** 2023-06-17

**Authors:** Gagandeep Lamba, Nilima R Thosar, Sandeep Khandaitkar, Samrudhi Khondalay

**Affiliations:** 1 Department of Pediatric and Preventive Dentistry, Sharad Pawar Dental College & Hospital, Datta Meghe Institute of Higher Education and Research (Deemed to be University), Wardha, IND; 2 Department of Pediatric and Preventive Dentistry, VSPM's Dental College and Research Centre, Nagpur, IND; 3 Department of Pediatric and Preventive Dentistry, Sharad Pawar Dental College and Hospital, Datta Meghe Institute of Medical Sciences (Deemed to be University), Wardha, IND; 4 Department of Oral and Maxillofacial Surgery, VSPM's Dental College and Research Centre, Nagpur, IND

**Keywords:** pediatric preventive dentistry, streptococcus mutans, early childhood caries, preventive dentistry, infant oral hygiene

## Abstract

Background

Dental caries is one of the most common oral health diseases in children. Early childhood caries (ECC) in children can lead to delayed overall growth in the future. This can be prevented by early initiation of infant oral hygiene practices (IOHP). As mothers are the primary caregivers, assessing their behavioral factors that play a significant role in IOHP can help design a customized health prevention plan for appropriate infant oral hygiene practices.

Methodology

A questionnaire based-study was carried out in an *Anganwadi* (rural child care center in India) of Nagpur region, Central India, for two months. Parents of young children were interviewed about the oral health care of children. The study was planned to evaluate the various methods used by parents to perform IOHP and to identify various behavioral determinants that affect IOHP. An integrative model of behavior change was used to evaluate factors determining the behavioral determinants in performing oral hygiene.

Results

Out of 144 parents, 105 (72.92%) initiated IOHP immediately after birth. Most of them (76, 52.78%) used a moist cloth to clean the oral cavity. The other methods used were the parent’s finger, water, etc. Most of the parents were unaware of commercially available oral wipes. Out of the various factors of behavioral determinants of integrative theory, oral health beliefs, emotional reactions, self-standard, and skills played a significant role in modifying parents’ intention to maintain the oral hygiene of young children. There was a direct correlation between the educational qualification of the mother and oral hygiene practices.

Conclusion

The results of this study reveal an integrative structure that includes factors like oral health beliefs, emotional reactions, self-standard, external support, social norms, and skills that are responsible for the behavior of parents towards oral health care. These factors vary from individual to individual. Modifying these specific behavioral determinants in parents could improve the oral hygiene practices of infants and toddlers. Community-based oral health care programs should be tailormade to target these specific barriers.

## Introduction

Early childhood caries (ECC) affects children and is a burden on the economy and public health. Dental caries in preschoolers could be linked to various factors such as diet, oral hygiene practices, and infant feeding practices. Preventive intervention like tooth brushing and infant oral hygiene practices (IOHP) remains the cornerstone of oral care and hence IOHP initiated soon after birth holds an important place for lifetime oral health [1]. These preschool years are crucial for lifelong health, including oral health behavior. Parental behavior, especially of mothers, plays an important role in the overall development and oral health care of the child [2]. Their active participation in IOHP can inculcate positive behavior related to oral health in young children. Thus, a good insight into these behavioral determinants is important to understand the infant oral hygiene practices followed by parents.

Various theories have been proposed that correlate the behavior and actions of individuals. Fishbein and colleagues have proposed an integrative model of health behavior that incorporates a number of well-known theories of behavior performance and behavior change. This model believes that intention is the primary determinant of behavior. The three factors governing strong intention are attitude to perform, perceived norms, and self-efficacy. The intention to perform a behavior could be impeded by external constraints or a lack of skills. Hence, an effective health promotion program can be tailor-made based on individual determinants of behavior [3]. Recent systematic reviews and meta-analyses have specified a need for prospective studies and trials that are based on various behavioral theories and evaluate the determinants of oral health behavior. Thus, it is necessary to identify these behavioral factors responsible for oral health care practices [4].

The role of these behavioral determinants is untapped by studies evaluating infant oral hygiene practices. The community characteristics like residing in a rural area, educational qualification, and exposure to social media could be important factors determining IOHP. So far, as per the authors' knowledge, no study has been done to evaluate the IOHP based on the behavioral determinants of an integrative model. Thus, the aim of this study was to recognize factors that support IOHP by parents. The study was planned to ask specific questions related to various methods used by parents to perform IOHP and to identify various behavioral determinants that affect IOHP.

## Materials and methods

The sample was a rural population located near an *Anganwadi* (rural child care center in India) in the central part of India. The study sample comprised 144 preschool children aged 0 to 24 months. The parents who participated in the study were of low socioeconomic status. The sample size was determined considering the proportion of parents having awareness about IOHP as the main outcome. The sample size was determined 144 based on the pilot study done on 10 respondents in the rural area and considering the expected proportion - 40%, relative precision - 20%, and desired confidence interval - 90%.

In order to involve parents and community-based health professionals like the Anganwadi workers in study design and data collection, we used a community-based participatory research approach. This method involved one-to-one interviews with parents who were invited to participate in the research and provide valuable information to determine the various methods used by them to perform IOHP and to identify various behavioral determinants that affect IOHP.

A pilot study was carried out to evaluate any changes that were required in the questionnaire, the questionnaire was further reviewed, modified, and finally approved by the Institutional Ethics Committee of** **VSPM's Dental College and Research Centre, Nagpur (approval number: VSPM/DCRC/IEC/TEA/2020/PEDODONTICS). The team conducting the study consisted of junior residents, who were trained by the principal investigator, to take one on one interviews. The community workers were also trained to explain to the uneducated mothers the main aim of the study and assist them in understanding the questions. Informed written consent was taken from parents just prior to the interview.

The interview included open-ended questions regarding whether parents practice oral hygiene for their children, and if they do so how they do it, when are these practices performed, and why did they initiate infant oral hygiene practices. To measure the parents’ intention towards IOHP, various factors were considered and correlated. The questions were grouped and assessed into the seven determinants of behavior based on integrative theory.

## Results

One hundred and forty-four parents willingly took part in this observational study - 133 (92.36%) were mothers and 11 (7.64%) were fathers. The parents' age ranged from 18 to 42 years, out of which 50 (34.72%) were first-time parents while 89 (61.81%) were second-time parents. There were five (3.47%) parents who had more than two children. The educational qualification of parents varied from primary education to post-graduate degree - 19 (13.19%) parents had primary education, 83 (57.64%) secondary education, 39 (27.08%) held graduate degrees, while only a few had post-graduation degrees. The children in the study sample included 78 (54.17%) girls and 66 (45.83%) boys. Their age range varied from 2 to 24 months, with 77 children below six months, 20 children six months to 12 months, and 47 children between 12 to 24 months. Based on the integrative model following observations were recorded.

Initiation and frequency of infant oral hygiene practices

**Figure 1:**
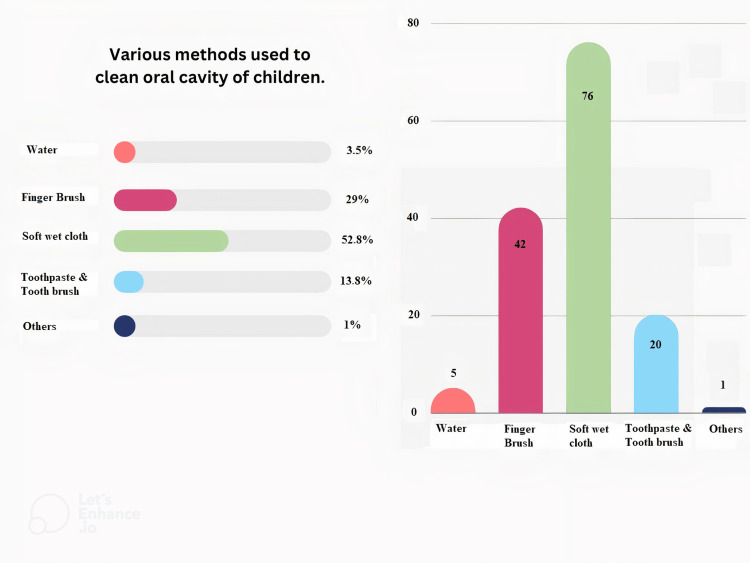
Various methods used to clean oral cavity of infants and young children

Out of 144 participants, a majority of 105 (72.92%) parents began cleaning the oral cavity as soon as the child was born. While 23 (15.97%) parents started cleaning the oral cavity after eruption of the first tooth, eight (5.56%) started cleaning after complete eruption of primary teeth and only a few (eight, 5.56%) parents were unable to provide any details for the same. Almost half, 76 (52.78%) parents, used soft wet cloth piece while 42 (29.17%) parents used their fingers to clean the oral cavity and remaining used either water or randomly cleaned the oral cavity as shown in Figure 1. Most of them were 113 (78.47%) unaware of commercially available tooth wipes.

Determinants of IOHP

**Figure 2:**
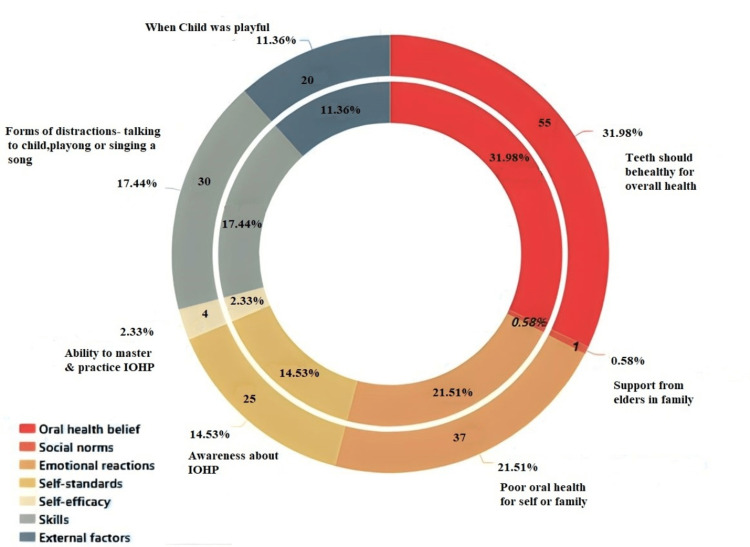
Behavioural determinants based on integrative model IOHP: infant oral hygiene practices

While interviewing about the reasons behind starting IOHP, parents discussed one or more of seven determinants mentioned in the integrative model. The most common behavioral determinants as shown in Figure 2 are - oral health beliefs, emotional reactions, self-standards, skills, self-efficacy, and external support.

Oral health beliefs

Most parents (55, 38.18%) believed that the oral hygiene of young children was important. They believed that the earlier initiation of IOHP resulted in the better outcome. It was apparent in their actions, as a large percentage of parents in our study had initiated infant oral hygiene immediately after birth. Parents mentioned that teeth should be healthy, as they affect overall health.

Emotional reactions

Thirty-seven parents (25.69%) cited emotional reactions. They mentioned that it was a source of motivation for them to maintain the oral hygiene of their children. Most of these parents themselves had bad dental experiences and hence did not want their child to suffer from dental caries.

Self-standards

Although, most parents were aware of the infant oral hygiene schedule, only a few (25, 17.36%) could follow the schedule of performing it twice daily. Most of the parents mentioned that following IOHP in the morning was easier compared to nighttime or twice daily. Hence nighttime was generally skipped. These parents tried their best to inculcate good oral hygiene practices.

Skills

Thirty parents described using various methods to engage their children in infant oral hygiene methods. Parents mentioned using the distraction method so that it was easier to clean the oral cavity. Parents also mentioned singing or turning toothbrushing into a game, so that children did not oppose while cleaning the oral cavity. A few parents mentioned using mobile phones to distract their child.

Self-efficacy

It is the ability to master and practice the schedule for infant oral hygiene. Only four parents actually considered it as a ritual and followed it very regularly.

External support

Twenty parents also mentioned that it was easier to perform infant oral hygiene if the child was in a good and playful mood. If child-friendly methods are used to maintain oral hygiene, acceptance might increase. Parents mentioned that an easy-to-use method that does not disturb the activity of the child could be the best way to maintain oral hygiene in young children.

Social norms

One parent out of 144 parents reported support from extended family. Thus, the mother was the prime caregiver in most of the cases.

When asked about the comfort of the child while brushing the teeth, 87 (60.42%) parents disagreed and they mentioned that cooperation was achieved if the child was distracted in different ways. Although most parents were aware that oral hygiene should be performed at least twice a day. There were only 26 (18.06%) who reached the goal - 96 parents (66.67%) missed it in the morning as well as at night. Also, nighttime practices were skipped frequently 47 (32.64%). Parents also reported that they received no information from dentists or other sources on how to clean the oral cavity. Table 1 shows the correlation between the mother’s education and IOHP, which was statistically significant (P=0.022).

**Table 1:** Association between maternal education status and IOHP IOHP: infant oral hygiene practices

Education of mother	IOHP		
	Yes - n (%)	No - n (%)	P-value = 0.022* Pearson chi^2 ^= 5.2618
Yes	104 (81.25%)	9 (56.25%)	
No	24 (18.75%)	7 (43.75%)	
Total	128	16	

A significant number of parents showed intention to change their current infant oral hygiene practices for the better, especially cleaning the oral cavity since birth (P=0.031), using moist cloth (P=0.046), using fluoridated toothpaste (P=0.011), and visiting dental clinic (P=0.033).

Multiple logistic regression analysis reveals the role of the education of the mother, knowledge of IOHP, first dental visit, and child’s age as significant factors for intention to change (Table 2). It indicated that knowledge about IOHP is the most significant predictor for intention to change. Mothers having correct knowledge about IOHP are almost three times (OR=2.82) more likely to change their intention if the rest of the factors like education, child’s age, etc. are adjusted. Similarly, the mother’s education is an important factor (OR=1.81) for intention to change, followed by the child’s age (OR=1.81) and knowledge of the first dental visit of the child (OR=1.78)*.*

**Table 2:** Multiple logistic regression analysis for predicting dichotomous outcome (intention to change) HSC: higher secondary certificate

Predictors for intention to change	Odds Ratio	95% Confidence Limits for OR Lower Upper		P-value
Education above HSC	1.81	0.76	4.32	0.18
Knowledge about infant oral hygiene practices	2.82	1.06	7.51	0.04
Knowledge about the first dental visit	1.78	0.64	4.97	0.27
Child’s age > 6 months	1.81	0.91	3.60	0.09

## Discussion

The main aim of the study was to evaluate the behavioral determinants responsible for IOHP in a rural area. We chose the rural community, as little is known about the IOHP of rural caregivers. The basis of IOHP is educating parents about oral health care and creating a disease-free oral cavity to build lifetime overall well-being [5]. *Streptococcus mutans *(SM) is known to be responsible for ECC. Improper oral hygiene during the early phase of life prevents the removal of biofilm and hence develops a caries-risk intraoral environment that can have high SM counts [6,7]. Earlier the colonization of SM, the higher the risk for ECC. Thus, initiating infant oral hygiene practices at an earlier age could prevent its colonization and maintain better oral hygiene, thus delaying or preventing ECC.

In our study, mothers were aware of the time of initiation of IOHP, 72.92% of mothers reported initiating IOHP immediately after birth and they started cleaning the oral cavity likewise. This signifies their knowledge of infant oral hygiene. Our results were similar to a study done by Suresh et al. and Mahmoud et al. who reported that 73.8% and 58.2% of mothers, respectively, had sufficient knowledge regarding IOHP [8,9]. On the contrary, another study reported that parents started infant oral hygiene practices quite late [10,11]. This variation in mothers’ knowledge could be related to varied education levels or varied exposure to social media or health educators.

While in our study parents (52.78%) were aware that soft cloth could be used to clean the oral cavity in infants prior to the eruption of teeth, a few (29.17%) used finger brush after the eruption of teeth. Similar results were observed in a study where parents were aware of different oral hygiene methods [12]. In our study, most of the parents (78.47%) were unaware of commercially available oral wipes that could be used for oral hygiene methods. Parents were also unaware of the other adjuncts that could be used with infant oral hygiene practices. Hence, parent education directed towards various age-appropriate oral hygiene aids available could be a beneficial approach to maintaining better oral hygiene.

It was surprising to know the main reason for performing infant oral hygiene practices in our study. Most (72%) of the parents performed IOHP to avoid bad breath in the children, while only a few reported that infant oral hygiene was performed to prevent caries in the future (13.19%). The other reasons cited for the same were advice from elders at home or on social media. This reveals that though the parents performed oral hygiene, they were unaware of the exact reasons why it should be done. Thus, educating parents directly or through the Anganwadi workers who are the first contact of parents in rural areas could prove beneficial in imparting proper knowledge. Dental awareness directed at why oral hygiene is important might bring about a transformation in the mindset of parents to initiate and maintain the oral hygiene of young children.

Nearly 66.67% of parents were aware that IOHP should be performed twice daily. But only 18% could follow the schedule. This is contrary to the data reported by Vargas et al. [13], where 65% of the parents performed brushing twice daily. Most of the times night time cleaning was skipped. The most probable reason cited by parents was that, by the end of the day, the child would be sleepy and did not cooperate for IOHP. Thus, one needs to target specific barriers faced by parents and tailor-made plans should be executed to overcome these barriers. An infant oral hygiene method that is comfortable to perform even when the child is asleep or cranky could be a better alternative rather than skipping the routine.

Most of the parents (60.42%) mentioned that the child did not cooperate while cleaning the oral cavity, but became cooperative if the child was distracted (77.78%). Parents used various distraction methods like narrating a story, bribing the child, etc. Thus, parental involvement plays an important role in infant oral hygiene practices. Contrary to our study, a study done by Shetty et al [14] reported that children were very happy while brushing their teeth. The probable reason could be early exposure to oral hygiene practices or consistency on the part of the parents. The socio-economic status or burden of household work could also play a significant role in the devotion of parents to IOHP.

Our study also revealed that 78% of parents did not receive any information about IOHP from either dentists or pediatricians or any form of social media. Only 22% of parents reported receiving information from social media regarding dental issues in children or IOHP. Contrary results were observed in a study of Nigerian mothers by Oredugba et al. and the study by Moslemi et al. in Iran, who reported that 53% and 77% of mothers received their knowledge from electronic media and television, respectively [15,16]. Thus, newspapers, other social media, or regular reminders through Anganwadi workers in India or professionals could be an alternative to spreading information in rural areas about infant oral hygiene and dental care in children.

Based on the integrative model, various factors that can influence behavior for a particular activity are oral health beliefs, social norms, emotional reactions, self-standards, self-efficacy, and skills. Our study revealed that oral health belief in parents that oral hygiene is important and the emotional reactions like the child being cranky while cleaning the teeth, followed by other factors such as skills, external factors, and self-efficacy played a significant role in altering the behavior of parents towards IOHP.

Thus, in our study, parents believed that the oral health of a child was important and there is a higher probability that these parents would instill a positive attitude towards dental behavior in children. The study population in our study performed regular oral health care for their young children [17] but nighttime oral hygiene maintenance was difficult due to a lack of child cooperation. This finding was contrary to the study of Chhabra and Chhabra [18] who reported assumptions of parents that primary teeth are less important, which could prove to be a hindrance to developing effective preventive protocols. Successful intervention could be directed towards the building of skills, i.e., a method that is easier to perform at nighttime. An alternative, such as an oral wipe, could be a better option that could be used to maintain oral hygiene even when the child is asleep.

In our study, the emotional reactions of the child also played a pivotal role in performing IOHP. The parents in general did not want to disturb the child for cleaning the oral cavity if the child was cranky or upset. Skills were another important factor to perform IOHP. Parents mentioned using various distraction techniques like singing, narrating a story, etc. while cleaning the oral cavity. Hence, parental determination to inculcate good oral hygiene practices could also be a driving force to maintaining good oral hygiene in children. Very few parents in our study thought that infant oral hygiene as a routine is an important aspect and should be practiced on a regular basis, i.e., self-efficacy. Awareness amongst parents regarding IOHP and how to perform them is the key to preventive strategies and initiating oral hygiene practices from a younger age

The community-based sample and implementing integrative behavioural theory model were the study's strong points. Among the limitations of this study was the self-reported questionnaire, which could be biased due to social acceptance and recall biases. Furthermore, there is a possibility of uncontrolled confounding and residual confounding which could negatively affect the causal inferences to be drawn from the findings. Further theory-based studies on oral health behavior in dentistry are suggested.

## Conclusions

The integrative model of behavior is known to predict behavior and the intention behind it. The findings of this study provide significant information about the various IOHPs performed in rural areas. These could be used in the future to formulate policies to promote oral hygiene in children. Based on these predictors of infant oral hygiene, efficient oral health educational plans and interventions can be formulated to enhance mothers' behavior to perform oral health hygiene. The various behavioral determinants that affected IOHP included oral health beliefs, social norms, skills of the parent, etc. based on the Integrative model.

The mothers can receive concise oral health information and instruction about oral health from health experts and from Anganwadi workers who are educated, as they are the first line of assistance in rural areas. However, more studies are needed to further justify the results of this study.
